# Adenosquamous carcinoma of the gallbladder: case report of a rare entity

**DOI:** 10.1093/jscr/rjaf555

**Published:** 2025-07-24

**Authors:** Sara El Ghaffouli, Mouna Khmou, Manal El Beyeg, Soumya El Graini, Rachida Latib, Youssef Mahdi, Basma El-Khannoussi

**Affiliations:** Department of Pathology of the National Institute of Oncology, Ibn Sina University Hospital Center, Allal Al Fassi Avenue, Rabat 10100, Morocco; Faculty of Medicine and Pharmacy, Mohammed V University, Mohamed Belarbi Alaoui Avenue, Rabat 10000, Morocco; Department of Pathology of the National Institute of Oncology, Ibn Sina University Hospital Center, Allal Al Fassi Avenue, Rabat 10100, Morocco; Faculty of Medicine and Pharmacy, Mohammed V University, Mohamed Belarbi Alaoui Avenue, Rabat 10000, Morocco; Department of Pathology of the National Institute of Oncology, Ibn Sina University Hospital Center, Allal Al Fassi Avenue, Rabat 10100, Morocco; Faculty of Medicine and Pharmacy, Mohammed V University, Mohamed Belarbi Alaoui Avenue, Rabat 10000, Morocco; Faculty of Medicine and Pharmacy, Mohammed V University, Mohamed Belarbi Alaoui Avenue, Rabat 10000, Morocco; Department of Radiology of the National Institute of Oncology, Ibn Sina University Hospital Center, Allal Al Fassi Avenue, Rabat 10100, Morocco; Faculty of Medicine and Pharmacy, Mohammed V University, Mohamed Belarbi Alaoui Avenue, Rabat 10000, Morocco; Department of Radiology of the National Institute of Oncology, Ibn Sina University Hospital Center, Allal Al Fassi Avenue, Rabat 10100, Morocco; Department of Pathology of the National Institute of Oncology, Ibn Sina University Hospital Center, Allal Al Fassi Avenue, Rabat 10100, Morocco; Faculty of Medicine and Pharmacy, Mohammed V University, Mohamed Belarbi Alaoui Avenue, Rabat 10000, Morocco; Department of Pathology of the National Institute of Oncology, Ibn Sina University Hospital Center, Allal Al Fassi Avenue, Rabat 10100, Morocco; Faculty of Medicine and Pharmacy, Mohammed V University, Mohamed Belarbi Alaoui Avenue, Rabat 10000, Morocco

**Keywords:** adenosquamous carcinoma, gallbladder, surgery, histopathology, case report

## Abstract

Adenocarcinoma constitutes 90%–95% of all malignant gallbladder (GB) neoplasms, making it the predominant subtype. In contrast, adenosquamous carcinoma (ASC) is significantly less common and comprises both glandular and squamous components. This subtype is more aggressive and has a worse prognosis than GB adenocarcinoma. Currently, complete surgical resection remains the cornerstone of treatment. In this paper, we present the case of a 56-year-old female diagnosed with moderately differentiated ASC of the GB, who underwent radical cholecystectomy, including the resection of liver segments IVb and V.

## Introduction

Gallbladder (GB) carcinoma is a rare malignancy, accounting for nearly half of all biliary tract cancers with bleak prognosis due to the absence of specific signs and symptoms, the organ's deep anatomical location, and late clinical presentation [1]. According to WHO classification, adenosquamous carcinoma (ASC) of the GB is a rare uncommon histological subtype of GB carcinoma (1%–5%), comprised of both glandular and squamous components [1–3]. These tumors demonstrate aggressive biological behavior, often extending to adjacent structures [4]. Herein, we present the case of a patient diagnosed moderately differentiated ASC of the GB treated with radical cholecystectomy including liver segments IVB, V.

## Case report

A 56-year-old woman with no previous medical history presented with a one-month history of pain in the right upper abdomen, accompanied by vomiting and unintentional weight loss during this period. Physical examination revealed a soft abdomen with tenderness in the right upper quadrant, and there were no palpable masses or lymphadenopathy. Routine hematological tests were normal, liver function test results were normal, but both alkaline phosphatase (PAL) and gamma-glutamyl transferase (GGT) levels were elevated (PAL: 169 UI/L, GGT: 287 UI/L).

**Figure 1:**
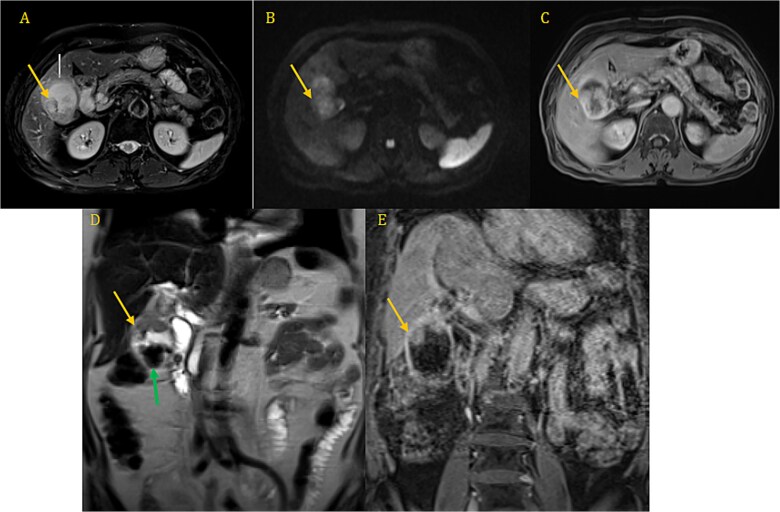
CP-MRI demonstrates markedly dilated gallbladder with cholelithiasis (green arrow) and wall thickening involving the superior portion and the infindubulum (yellow arrow) in iso-signal on T2 (A, D), restrictive on DWI (B), with a late enhacement after injection (C, E).

Both the computed tomography (CT) scan of the abdomen and pelvis and the magnetic resonance imaging (MRI) revealed cholelithiasis and irregular thickening of the GB wall (Fig. 1A–E).

**Figure 2:**
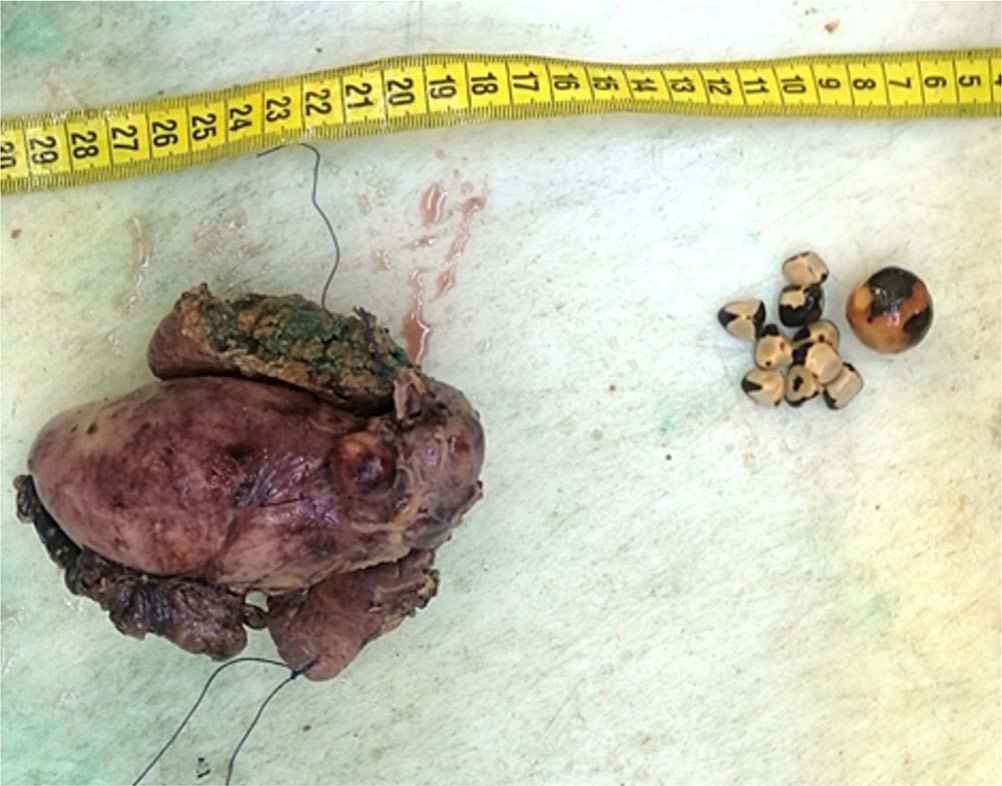
Macroscopic image of cholecystectomy including liver segment.

This patient was treated with a radical cholecystectomy including liver segments IVb, V, and lymph nodes dissection (Fig. 2). The specimens were sent for histopathological examination in formalin.

**Figure 3:**
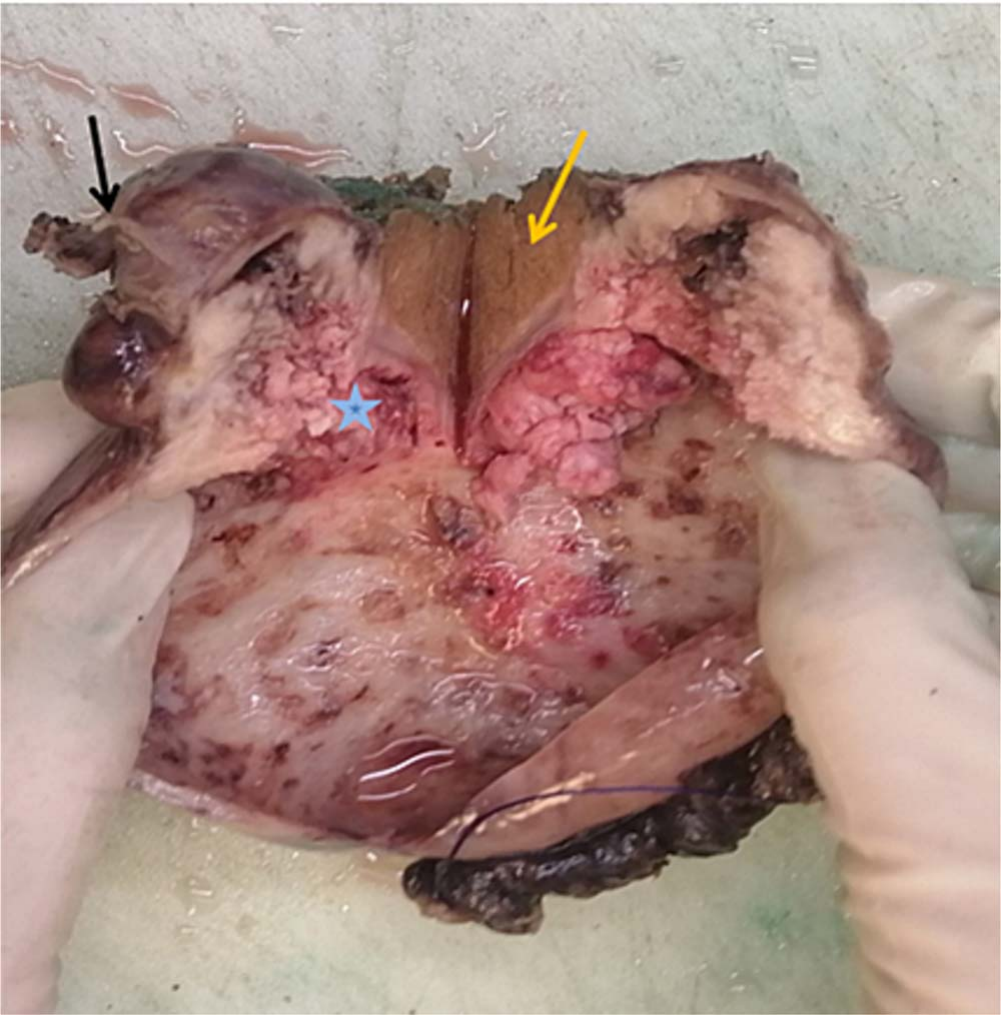
Macroscopic image of cut section showing the liver segment (yellow arrow), neck of the gallbladder (black arrow) and mass (blue star).

On gross pathological examination, the GB measured 11 × 10.5 × 5.5 cm; the hepatic segment measured 10.5 × 3 × 2.7 cm (Fig. 3).

The cut section revealed a mass measuring 4.7 × 2.6 × 2.5 cm involving the liver, also a well-defined, whitish nodule located 0.8 cm from the liver margin and 2.5 cm from the GB.

**Figure 4:**
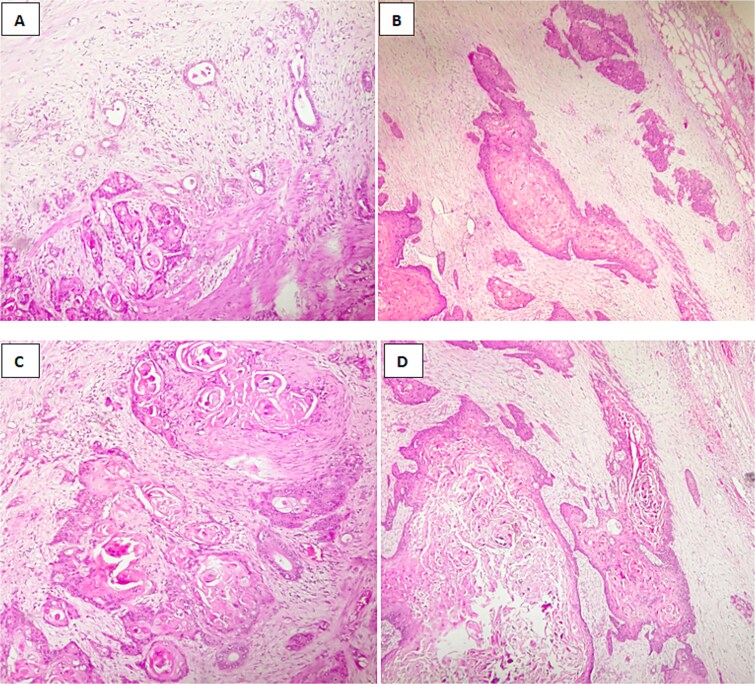
Histological image of adenosquamous carcinoma of the gallbladder showing both glandular and squamous components, with evident areas of keratinization. (A, B) H&E stain, ×100; (C, D) H&E stain, ×200.

Microscopic findings revealed a squamous component arranged in nests, clusters with keratin pearls;consists of atypical polygonal cells with intercellular bridges, showing, high nuclear to cytoplasmic ratio, irregular nuclear rim, prominent nucleoli, and abondant eosinophilic cytoplasm,a glandular component consisted of mucinous cells,with lymphovascular and perineural invasion, and tumor extension into adjacent liver (Fig. 4A–D).

Histopathological analysis of the lymph node dissection revealed three positive lymph nodes out of the seven that were removed, resulting in a total of three out of seven positive lymph nodes.

## Discussion

GB carcinoma is a rare and aggressive cancer. It affects individuals across all ethnicities and regions, but its incidence is highest in Northern India, Pakistan, East Asia, Eastern Europe, and parts of South America, including Colombia and Chile [3–5]. Risk factors for developing GB carcinoma include chronic inflammation, gallstones, porcelain GB, GB polyps, primary sclerosing cholangitis, chronic infections caused by Salmonella or *Helicobacter pylori*, congenital biliary cysts, and abnormal pancreaticobiliary duct junctions [3–6].

In the evaluation of GB pathologies, ultrasound is typically the first imaging choice due to its accessibility and effectiveness in detecting common abnormalities. However, for cases where malignancy is suspected, further staging workup is necessary to assess both local and distant spread. This often involves more detailed imaging modalities such as a CT scan of the abdomen and pelvis and MRI [3–13].

CT and MRI are valuable in determining the resectability of a tumor, as well as for assessing the tumor's spread. Specifically, GB carcinoma can present in various forms, including:

- A mass replacing the GB in 40%–65% of cases.
- Focal or diffuse GB wall thickening in 20%–30% of cases.
- An intraluminal polypoid mass in 15%–25% of cases.

MRI is particularly useful for evaluating more complex structures, such as the involvement of the hepatoduodenal ligament, portal vein encasement, and the presence of lymph node involvement [3–13].

The tumor, node, metastasis staging system of the combined American Joint Committee on Cancer and the Union for International Cancer Control is now the preferred method for classifying GB cancer. Staging is also influenced by the proliferative index of the tumor, as a higher proliferative index, particularly in squamous components of ASC, may correspond to a higher T (tumor) stage, indicating more advanced disease [3–13].

GB malignancies originate from the epithelium of the GB wall and can be categorized into adenocarcinoma, squamous cell carcinoma (SCC), or ASC based on histopathology [2]. Adenocarcinoma is the most common subtype, representing ~90%–95% of all cases. ASC consists of both glandular and squamous components, with the squamous component making up 25%–99% [1]. Tumors with <25% squamous components are referred to as adenocarcinomas with focal squamous change, while those lacking glandular components are classified as pure SCC. ASC and pure SCC together account for ~5% of all GB cancers [3–7].

The treatment of GB carcinoma is challenging and depends on the cancer stage, the patient's performance status, and the side effects of treatment [3–4]. However, therapeutic options are limited due to the aggressive nature of the disease. Curative surgical resection, such as cholecystectomy, is possible for patients with minimal invasion into local organs, where the tumor is confined to the gallbladder wall without lymph node or adjacent structure involvement in the hepato-duodenal ligament [3–8]. Early-stage tumors with liver invasion can be managed with liver resection alongside cholecystectomy, followed by systemic or regional chemotherapy. Studies have shown that patients undergoing radical tumor resection have better outcomes compared to those undergoing primary tumor resection alone [3–9]. Adenosquamous and SCC of the GB tend to be more amenable to resection than adenocarcinoma. Unfortunately, patients in advanced stages, where curative resection is not an option, derive minimal benefit from surgical resection and systemic chemotherapy. Recently, newer treatment approaches such as targeted therapy and chemoradiation have been explored for GB carcinoma patients [3, 8, 9].

It is uncertain whether the risk factors for the development of ASC are similar to those for adenocarcinoma [1, 2]. The prognosis for patients with adenosquamous or SCC of the GB remains poor, even after surgical resection. This is largely due to the tumor's histological grade and stage, which are associated with a higher rate of liver metastasis at the time of surgery compared to adenocarcinoma, as well as an increased likelihood of local lymph node involvement at diagnosis [1, 2, 10, 11]. The overall 5-year survival rate for ASC is < 5%, with a median survival of <6 months [3–12].

## Conclusion

ASC of the GB is an uncommon and aggressive form of GB cancer that has a worse prognosis than GB adenocarcinoma. At present, surgical intervention is regarded as the gold standard treatment. However, given the limited available literature on this rare condition, additional research is essential to develop a more tailored management strategy.
